# RNAi gene knockdown in the poultry red mite, *Dermanyssus gallinae* (De Geer 1778), a tool for functional genomics

**DOI:** 10.1186/s13071-020-04562-9

**Published:** 2021-01-18

**Authors:** Wan Chen, Kathryn Bartley, Francesca Nunn, Alan S. Bowman, Jeremy M. Sternberg, Stewart T. G. Burgess, Alasdair J. Nisbet, Daniel R. G. Price

**Affiliations:** 1 Moredun Research Institute, Pentland Science Park, Bush Loan, Penicuik, Midlothian EH26 0PZ UK; 2 Institute of Biological and Environmental Sciences, School of Biological Sciences, University of Aberdeen, Aberdeen, AB24 3FX UK

**Keywords:** RNA interference, Poultry red mite, Functional genomics, Gene silencing, Gene knockdown

## Abstract

**Background:**

The avian haematophagous ectoparasite *Dermanyssus gallinae*, commonly known as the poultry red mite, causes significant economic losses to the egg-laying industry worldwide and also represents a significant welfare threat. Current acaricide-based controls are unsustainable due to the mite’s ability to rapidly develop resistance, thus developing a novel sustainable means of control for *D. gallinae* is a priority. RNA interference (RNAi)-mediated gene silencing is a valuable tool for studying gene function in non-model organisms, but is also emerging as a novel tool for parasite control.

**Methods:**

Here we use an *in silico* approach to identify core RNAi pathway genes in the recently sequenced *D. gallinae* genome. In addition we utilise an *in vitro* feeding device to deliver double-stranded (ds) RNA to *D. gallinae* targeting the *D. gallinae vATPase subunit A* (*Dg vATPase A*) gene and monitor gene knockdown using quantitative PCR (qPCR).

**Results:**

Core components of the small interfering RNA (siRNA) and microRNA (miRNA) pathways were identified in *D. gallinae*, which indicates that these gene silencing pathways are likely functional. Strikingly, the P-element-induced wimpy testis (PIWI)-interacting RNA (piRNA) pathway was absent in *D. gallinae*. In addition, feeding *Dg vATPase A* dsRNA to adult female *D. gallinae* resulted in silencing of the targeted gene compared to control mites fed non-specific *lacZ* dsRNA. In *D. gallinae*, dsRNA-mediated gene knockdown was rapid, being detectable 24 h after oral delivery of the dsRNA, and persisted for at least 120 h.

**Conclusions:**

This study shows the presence of core RNAi machinery components in the *D. gallinae* genome. In addition, we have developed a robust RNAi methodology for targeting genes in *D. gallinae* that will be of value for studying genes of unknown function and validating potential control targets in *D. gallinae*. 
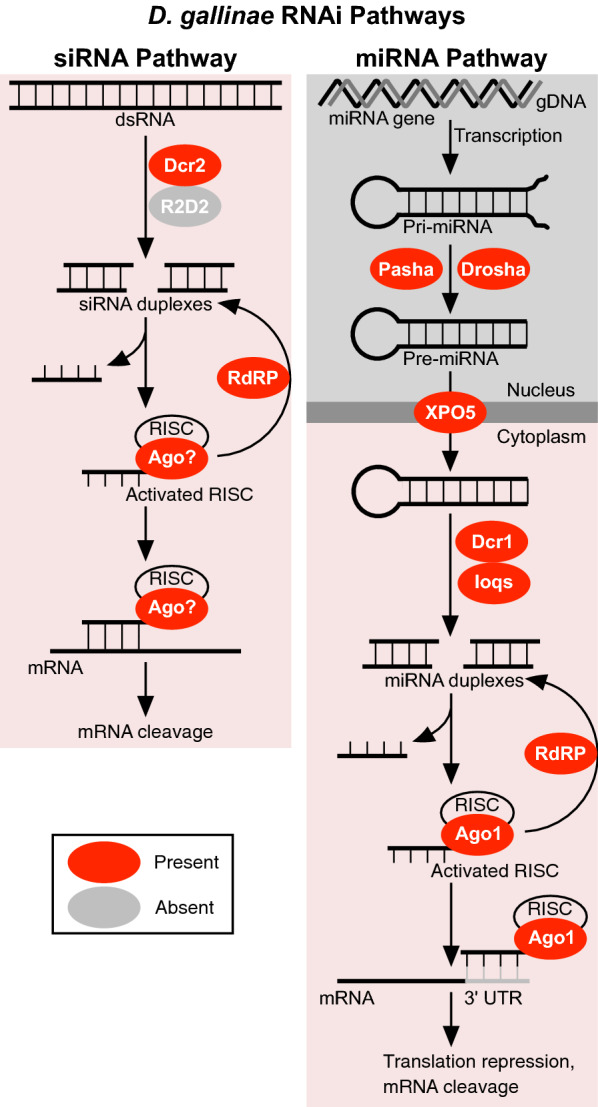

## Background

Poultry red mite [*Dermanyssus gallinae* (De Geer, 1778)]*,* is an avian haematophagous ectoparasite with a worldwide distribution and a prevalence of 83% in European hen egg-laying facilities [1]. There are five life-cycle stages in *D. gallinae*: egg, larvae, protonymph, deutonymph and adult, with blood-feeding being a feature of only the latter three stages [2, 3]. This parasite lives off-host in the cracks and crevices in the hen facilities and only emerges to bite the host in darkness for a blood meal, which takes around 30–90 min, during which each mite consumes approximately 200 μg of blood per feed [3, 4]. Thus, in severe infestations where each laying hen may be infested with up to 500,000 mites, infestation can lead to multiple behavioural and physiological changes in the birds, such as restlessness, irritation, anaemia, feather pecking, cannibalism and increased mortality rates [5, 6]. Also, *D. gallinae* has been reported to be the vector for a number of bacterial and viral diseases of birds as well as zoonotic agents [7, 8]. Apart from the hen welfare issues caused by *D. gallinae*, infestation by this mite also increases the operational expenditure for hen egg production through losses in feed conversion ratio, downgrading of eggs and decreased egg output [6, 9, 10]. Overall, the estimated annual cost of *D. gallinae* (production loss plus costs of control) was estimated to be €231 million in Europe in 2017 [6]. Conventional control of *D. gallinae* is through the use of chemical acaricide treatments of poultry houses or through systemic acaricides administered in the drinking water [11]. However, with the incidence of resistance against some acaricides increasing [12] and concerns over residues in food, multiple chemical treatments have been withdrawn from use in the European Union [2].

The development of novel strategies for control of *D. gallinae* is a priority, and the identification of gene targets that will faciliate the development of novel control approaches has been facilitated by the recent publication of transcriptomes and the draft genome of *D. gallinae* [13, 14]. One key tool for exploiting these genomic and transcriptomic resources for novel target identification is transcriptional silencing. Since the discovery of RNA interference (RNAi) as a tool for silencing gene expression in the free-living nematode, *Caenorhabditis elegans* [15], RNAi application has been widened into various fields, including novel arthropod control strategies [16, 17]. Irrespective of the organism in which transcriptional silencing is to be investigated, two essential components are required for successful RNAi: (i) the presence of a functional RNAi pathway; (ii) an appropriate delivery method for the gene-specific double-stranded RNA (dsRNA) to initiate the silencing process. RNAi-mediated gene silencing in mites was first demonstrated in the two-spotted spider mite *Tetranychus urticae* [18] and since this initial discovery RNAi pathways have been reported in other mite species (reviewed in [19]). For several mite species, delivery of dsRNA has been achieved through soaking mites in solutions containing the dsRNA (e.g. see [20, 21]). While the immersion method of dsRNA delivery has also caused gene knockdown in *D. gallinae* [22], high mortality rates were observed, thus hampering interpretation of transcriptional silencing data and necessitating the development of a better approach of dsRNA administration to *D. gallinae*.

In the study reported here, we use the recently published *D. gallinae* draft genome [13] and associated transcriptomic data to describe the RNAi pathway in *D. gallinae* and to investigate an optimised delivery method to ascertain the optimal properties of the dsRNA for RNAi in this species. RNAi-mediated gene silencing was investigated in adult female *D. gallinae* mites by targeting *D. gallinae vacuolar ATPase subunit A* (*Dg vATPase A*), which has previously been targeted in arthropods, including the two-spotted spider mite *Tetranychus urticae* [23, 24]*.*

## Methods

### RNAi pathway gene annotation

Core RNAi pathway components were identified in the *D. gallinae* genome by sequence similarity to RNAi pathway genes from *T. urticae* [25] and *Drosophila melanogaster*. Core RNAi components were selected to represent three RNAi pathways, namely the microRNA (miRNA) pathway (*Argonaute-1*, *Dicer-1*, *loquacious*, *Drosha*, *Pasha*, *Exportin-5*), the small interfering RNA (siRNA) pathway (*Argonaute-2*, *Dicer-2*) and the P-element induced wimpy testis (Piwi)-interacting RNA (piRNA) pathway (*Aubergine*, *Piwi*, *Argonaute-3*); and also RNA-directed RNA polymerase (RdRP), which is required for amplification of the RNAi silencing signal. Amino acid sequences for all 12 *T. urticae* RNAi pathway components were retrieved from Ensembl Genomes [26] and used as query for BlastP (Protein–protein BLAST [Basic Local Alignment Search Tool]) searches against predicted peptides from the *D. gallinae* genome [13]. Identified *D. gallinae* RNAi components were considered sequence orthologues for a given RNAi pathway component when they met the criteria of reciprocal best hit and included one-to-one and one-to-many sequence orthologues. Functional domains were identified in retrieved *D. gallinae* sequences by searching the Pfam database [27].

### Phylogenetic analysis

For the phylogenetic analysis, selected argonaute protein sequences were aligned using the multiple alignment program MUSCLE [28]. Short sequences (< 50% of the protein’s consensus length) and predicted non-functional sequences due to absence of either the PAZ domain (PF02170) or the Piwi domain (PF02171) were removed from the alignment. All sequences used for phylogenetic reconstruction are shown in Additional file 1: Table S1. Ambiguously aligned positions were excluded by the trimAL v1.2 program [29], and a maximum-likelihood (ML) phylogenetic tree was constructed in MEGA 10.1.8 [30] using a LG+G substitution model. Statistical tree robustness was assessed using bootstrap analysis (1000 bootstrap replicates).

### Amplification of *D. gallinae vATPase A* gene for RNAi validation.

The full-length sequence of *D. gallinae vacuolar ATPase subunit A* (*Dg vATPase A:* DEGAL4806g00010) was retrieved from the ORCAE database for *D. gallinae* [13, 31]. To validate the ORCAE gene model, *Dg vATPase A* (DEGAL4806g00010) was used in a Blastx search against the National Center for Biotechnology Information (NCBI) non-redundant protein database, and sequences with high similarity were retrieved from *Varroa destructor* (XP_022670783 and XP_022670784), *Tropilaelaps mercedesae* (OQR76956), *Galendromus occidentalis* (XP_003741079) and *Ixodes scapularis* (XP_029849202). All sequences were aligned using MUSCLE [28], and primers were designed based on conserved regions across *Dg vATPase A* and all other aligned sequences. The Clustalx alignment and region used for primer design is shown in Additional file 2: Figure S1. The full-length coding sequence of *Dg vATPase A* was amplified using female *D. gallinae* cDNA as template and verified by Sanger sequencing.

### dsRNA Synthesis

Region 1 (R1: 495 bp, corresponding to exon 4–7) and region 2 (R2: 385 bp, corresponding to exon 8) of the *Dg vATPase A* gene were amplified from cDNA generated from adult female *D. gallinae* using Phusion proof-reading polymerase (Thermo Fisher Scientific, Waltham, MA, USA). Each forward and reverse primer contained an NcoI and NheI restriction enzyme site, respectively, to allow directional cloning into the RNAi vector pL4440 (pL4440 was a gift from Andrew Fire [Addgene plasmid # 1654; http://n2t.net/addgene:1654). Primer sequences are shown in Additional file 3: Table S2. Amplification products for *Dg vATPase A* R1 (495 bp) and R2 (385 bp) were digested with* Nco*I and* Nhe*I and cloned into the corresponding restriction enzyme sites of pL4440. Plasmids were used to transform chemically competent *Escherichia coli* JM109 cells (Promega, Madison, WI, USA), and plasmid was isolated from *E. coli* transformants using a Wizard® *Plus* SV Minipreps DNA Purification System (Promega). Both RNAi constructs containing *Dg vATPase A* R1 and R2 were verified by Sanger sequencing. For control (non-target) dsRNA production we used a previously generated construct containing a region of the *E. coli* strain K-12 *lacZ* gene NC_000913 (319 bp; 63–381 bp of the CDS) cloned into the SacI and SmaI sites of pL4440 [20]. dsRNA was synthesized using the T7 RiboMAX Express RNAi System (Promega), according to the manufacturer’s instructions. For RNA synthesis, *Dg vATPase A* pL4440 plasmids were linearised with either NcoI or NheI for sense or antisense transcription, respectively. Control *lacZ* pL4440 plasmid was linearised with SmaI or BglII for sense or antisense transcription, respectively. For dsRNA production, equimolar amounts of complementary RNAs were mixed and incubated at 70 °C, then slowly cooled to room temperature. Annealed dsRNAs were purified by sodium acetate/isopropanol precipitation, resuspended in nuclease-free water and quantified on a NanoDrop One spectrophotometer (Thermo Fisher Scientific). Annealed dsRNAs were analysed by agarose/TAE (Tris-acetate–EDTA [ethylenediaminetetraacetic acid]) gel electrophoresis to confirm quality and predicted size.

### siRNA synthesis

*Dg vATPase A* siRNAs were synthesized by either *in vitro* digestion of long dsRNAs (method 1) or by chemical synthesis of 27-mer blunt dsRNAs (method 2). In method 1, long dsRNAs for R1 and R2 of the *Dg vATPase A* gene and *lacZ* control gene (120 μg of each dsRNA) were incubated with 0.2 units/μl ShortCut® RNase III (New England BioLabs, Ipswich, MA, USA) for 3 h at 37°C to produce a heterogeneous mix of short (18–25 bp) siRNAs. Reactions were stopped with EDTA according to the manufacturer’s protocol and precipitated in ethanol, following which the size distribution of the digested RNAs was validated by electrophoresis using a 4% agarose gel. In method 2, Dicer-substrate siRNAs (27-mer blunt dsRNAs) were designed based on the coding sequence of the *Dg vATPase A* gene and *lacZ* control gene using the Eurofins Genomics siMAX siRNA design tool, commercially synthesized and then annealed by Eurofins Genomics (Eurofins Genomics, Ebersberg, Germany). The sequence of each siRNA is shown in Additional file 4: Figure S2.

### RNAi feeding trials

*Dermanyssus gallinae* mites were collected, with sampling including a mixture of stages and sexes, from commercial egg-laying facilities and stored in vented 75-cm^2^ tissue culture flasks (Corning Inc, Corning, NY, USA). For optimal *in vitro* feeding, the collected mites were conditioned at room temperature for 7 days, after which they were stored at 4 °C for 3 weeks without access to food, according to [32]. For oral delivery of dsRNA and siRNA to *D. gallinae* mites, approximately 100 mites were housed in an *in vitro* feeding device constructed as described previously [33]. Each replicate feeding device contained 200 µl of freshly collected heparinised goose blood (20 units/ml) with dsRNA at concentrations described in each experiment. For each dsRNA feeding experiment, biological replicates consisted of an independent group of mites in replicate feeding devices (*n* = 3–6, depending on the experiment). Feeding devices were placed in a MLR-351H relative humidity incubator (SANYO, Moriguchi, Japan) for 3 h at 39 °C to initiate mite feeding, followed by 21 h at 25 °C for mite recovery, both at 85% relative humidity. After 24 h, fed adult female mites were recovered from each replicate feeding device and transferred to separate labelled 1.5-ml tubes, which were kept at 25 °C in dark conditions for the duration of the experiment. Mites from each replicate group were flash-frozen in liquid nitrogen at the time-points indicated in each experiment and stored at − 70 °C for later RNA extraction.

### Quantitative real-time PCR analysis

Real-time quantitative PCR (qPCR) was used to quantify *Dg vATPase A* gene expression in adult female mites from RNAi feeding trials. Mites were homogenised with a tube pestle, and total RNA was isolated using an RNeasy^®^ Plus Micro Kit (Qiagen, Hilden, Germany) equipped with a genomic DNA (gDNA) eliminator spin-column. Total RNA was quantified using a NanoDrop One spectrophotometer (Thermo Fisher Scientific), and first-strand complementary DNA (cDNA) was synthesized using a QuantiTect® Reverse Transcription Kit (Qiagen), according to the manufacturer’s protocol.

qPCR primers were designed for the target gene *Dg vATPase A* (DEGAL4806g00010) and the housekeeping reference gene coding glyceraldehyde 3-phosphate dehydrogenase (*GAPDH*; DEGAL4146g00090) using Primer3Plus [34]; the primer sequences are shown in Additional file 3: Table S2. Primers were checked for specificity by alignment of *D. gallinae* target sequences with goose *Anser cygnoides v-ATPase A* (XM_013196364) and *GAPDH* (XM_013199522). For construction of standard curves, qPCR primers were used to amplify *Dg vATPase A* (DEGAL4806g00010) and *GAPDH* (DEGAL4146g00090) from adult female *D. gallinae* cDNA. Amplification products were cloned into vector pJET1.2 (Thermo Fisher Scientific) and verified by DNA sequencing; amplification was specific for *Dg vATPase A* and *D. gallinae GAPDH*. Plasmids were used in qPCR experiments to construct standard curves from 10^2^–10^8^ copies of each gene. qPCR reactions were carried out in a volume of 10 μl comprising 1× PowerUp SYBR Green Master Mix (Thermo Fisher Scientific), 500 nM of forward and reverse primers and cDNA derived from 1 ng total RNA for each sample. PCR reactions were performed on an Applied Biosystems 7500 Real Time PCR System (Applied Biosystems, Foster City, CA, USA); thermal cycling conditions were 50 °C, 2 min; then 95 °C/2 min; followed 95 °C/15 s, 58 °C/15 s, 72 °C/1 min for by 40 cycles. *Dg vATPase A* gene expression was normalised to housekeeping gene *GAPDH*, and expression levels were reported relative to control (*lacZ*) dsRNA-fed mites. qPCR experiments were performed in triplicate and included no template controls and no reverse transcription controls with each run.

### Statistical analyses

Analysis of *Dg vATPase A* gene expression levels in RNAi feeding trials were performed using GraphPad Prism version 8.0.0 for Windows (GraphPad Software, La Jolla, CA, USA). Datasets were analysed using either Student’s t-test or one-way analysis of variance (ANOVA) with Dunnett’s multiple comparison test (as indicated). *P* values of  < 0.05 were considered to indicate significance.

## Results

### miRNA and siRNA pathways are present in the *D. gallinae* genome

**Fig. 1:**
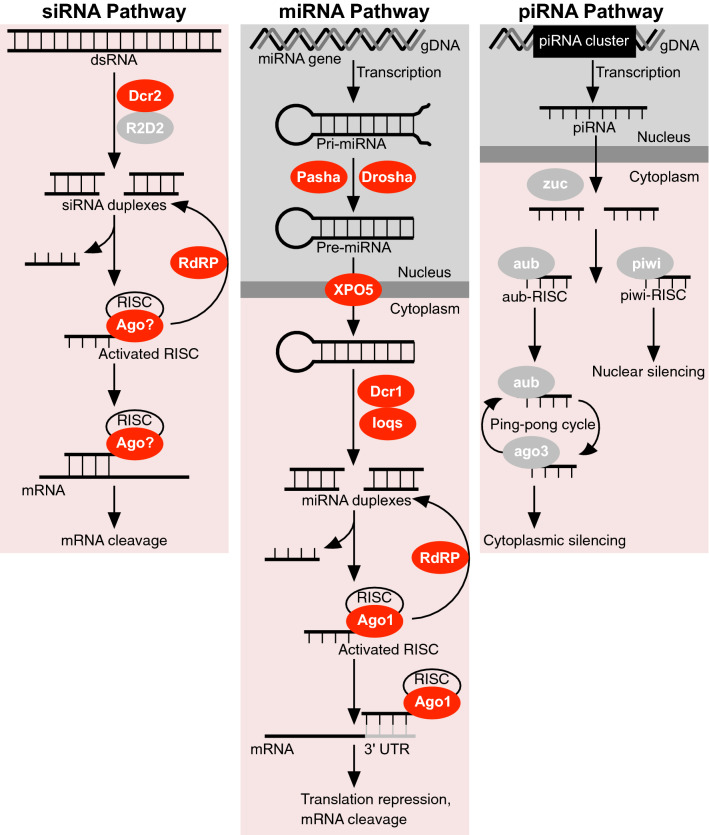
Summary of RNA interference (*RNAi*) pathways identified in *Dermanyssus gallinae*. RNAi pathways are based on those described in *Tetranychus urticae* and *Drosophila melanogaster* with core RNAi pathway enzymes either present (red) or absent (grey) in *D. gallinae*. siRNA pathway: double-stranded RNA (*dsRNA*; either viral or experimentally introduced) is processed by Dicer-2 (*Dcr-2*) into 21- to 23-nucleotide small interfering RNAs (*siRNAs*) and loaded into the RNA-induced silencing complex (*RISC*) complex. Argonaute (*Ago*) cleaves the passenger strand of the siRNA and retains the guide strand which guides the active siRISC complex to the target mRNA. Full complementarity between the guide siRNA strand and target mRNA leads to cleavage of the mRNA. miRNA pathway: The microRNA (*miRNA*) gene is transcribed in the nucleus to generate pri-miRNA that is then cleaved by the enzymes Drosha and Pasha to form a pre-miRNA. The pre-miRNA is transported to the cytoplasm through Exportin-5 (*XPO5*) and cleaved by Dicer-1 (*Dcr-1*)/Loquacious (*loqs*) complex to yield miRNA. The miRNA is loaded into the RISC complex, and Ago cleaves the passenger strand of the miRNA and retains the guide strand which guides the active miRISC complex to the target mRNA. Partial complementarity between the guide strand and target mRNA leads to either translation repression or cleavage of the mRNA. miRNAs usually target several genes, with shared sequences in the 3′ untranslated region (*UTR*). In both pathways siRNAs and miRNAs are amplified by the RNA-dependent RNA polymerase (*RdRP*). piRNA pathway: The P-element-induced wimpy testis (*piwi*)-interacting RNA (*piRNA*) pathway functions in germline cells to protect against transposable elements. Antisense piRNAs are transcribed from repetitive elements in genomic DNA (*gDNA*) and processed by zucchini (*zuc*) into 26- to 32-nucleotide primary piRNAs. Primary piRNAs associate with either piwi or aubergine (*aub*). Piwi-associated piRNAs are translocated to the nucleus, while aub-associated piRNAs cleave cytoplasmic transposon transcripts and trigger a ‘ping-pong’ piRNA amplification. Following transposon, transcript cleavage argonaute-3 (*ago3*) is loaded with secondary piRNAs which in turn produce piRNAs that associate with aub, resulting in silencing of cytoplasmic transposon transcripts

We used a systematic search for core RNAi genes involved in the siRNA pathway (*Argonaute-2*, *Dicer-2*), miRNA pathway (*Argonaute-1*, *Dicer-1*, *loquacious*, *Drosha*, *Pasha*, *Exportin-5*) and piRNA pathway (*Aubergine*, *Piwi*, *Argonaute-3*) in *D. gallinae*. Our searches identified *D. gallinae* orthologues of at least one core gene in the siRNA and miRNA pathways (See both Fig. 1 and Table 1) and did not identify piRNA pathway orthologues, suggesting that this latter pathway is not present in *D. gallinae*.

**Table 1.** Identification of *Dermanyssus gallinae* core RNA interference pathway genes

Gene name (gene symbol)	*Tetranychus urticae* accession	*D. gallinae* ortholog/s gene accession	(Gene #); top BLAST E-value
Dicer (*Dcr*)	tetur19g00520; tetur07g00990	DEGAL4207g00210; DEGAL2576g00010	(2); 0
Partner of Drosha (*Pasha*)	tetur36g00220; tetur36g00250	DEGAL6243g00040	(1); 1.00E–105
Drosha (*Drosha*)	tetur12g00910	DEGAL3563g00160	(1); 0.0
Loquacious (*loqs*)	tetur13g00430; tetur13g00410	DEGAL6165g00020^e^; DEGAL6165g00030^e^	(2); 5.00E–20
Argonaute (*AGO*)	tetur20g02910; tetur09g00620; tetur09g03140; tetur02g10560; tetur02g10580; tetur04g01190; tetur02g10570	DEGAL5747g00010; DEGAL5147g00020^c^; DEGAL2433g00050; EGAL1807g00010; DEGAL5891g00010; DEGAL2763g00020; DEGAL2329g00030; DEGAL3376g00010; DEGAL1832g00030; DEGAL3253g00060^b^; DEGAL3253g00070^b^; DEGAL5243g00010; DEGAL3253g00080^b^; DEGAL3253g00100^b^; DEGAL867g00020^g^; DEGAL5130g00010; DEGAL4347g00070; DEGAL1695g00010^a^; DEGAL1695g00020^a^; DEGAL4104g00020; DEGAL867g00010^g^; DEGAL6462g00020^f^; DEGAL6462g00010^f^; DEGAL2892g00010; DEGAL5147g00040^c^	(25); 0
P-element induced wimpy testis (*Piwi*); Aubergine (*aub*); Argonaute-3 (*AGO3*)	tetur06g03300; tetur28g00450; tetur28g00340; tetur06g05580; tetur06g05570; tetur06g05600; tetur17g03380	N/A	(0); N/A
Exportin-5 (*Exportin-5*)	tetur02g00520; tetur02g00500	DEGAL4407g00370	(1); 3.00E–28
RNA-directed RNA polymerase (*RdRP*)	tetur02g08750; tetur02g08780; tetur02g08810; tetur02g08820	DEGAL1833g00010; DEGAL2592g00050; DEGAL4182g00030; EGAL2262g00020; DEGAL6675g00010; DEGAL6621g00090; DEGAL6161g00150^d^; GAL3284g00080; DEGAL6161g00170^d^	(9); 1.00E–95

BLAST, Basic local alignment search tool; N/A, not available

^a–g^Each identified *D. gallinae* gene is an orthologue of *T. urticae* RNAi pathway genes based on best reciprocal BLAST hit. Genes located on the same *D. gallinae* scaffold are highlighted using the superscript letters a-g.

### siRNA Pathway genes

Searches were conducted to identify core siRNA pathway genes in *D. gallinae* based on similarity to *Argonaute-2* and *Dicer-2* from *T. urticae* and *Drosophila.* Using these searches we identified an orthologue of *Dicer-2* in *D. gallinae* (DEGAL2576g00010; Table 1), while our searches did not identify a *D. gallinae Argonaute-2* orthologue.

Dicer-2 (Dcr-2) is required to cleave and yield a mature siRNA. Domain analysis of *D. gallinae* Dcr-2 revealed a similar domain architecture to the well-characterised *Drosophila* Dcr-2, although the PAZ domain was absent in *D. gallinae* Dcr-2 (Additional file 5: Figure S3). Gene expression data available through ORCAE (Online Resource for Community Annotation of Eukaryotes) shows that *D. gallinae Dcr-2* is universally expressed in adult male and female mites and all other life-stages.

### miRNA Pathway genes

We identified orthologues of all six core miRNA pathway genes in *D. gallinae*. The identified *D. gallinae* miRNA pathway genes included *Drosha* (DEGAL3563g00160; Table 1) and *Pasha* (DEGAL6243g00040; Table 1), both required for miRNA biosynthesis; *Exportin-5* (DEGAL4407g00370; Table 1), required for the export of pre-miRNA from the nucleus to cytoplasm; *Dicer-1* (DEGAL4207g00210; Table 1) and two copies of its binding partner *Loquacious* (DEGAL6165g00020; DEGAL6165g00030; Table 1)], required to cleave and yield a mature miRNA; and *Argonaute-1* (DEGAL5747g00010; DEGAL5147g00020; Table 1), required to target and slice complementary RNA transcripts.

Domain analysis of *D. gallinae* Dcr-1 (Additional file 5: Figure S3) and *Argonaute-1* orthologues (Additional file 6: Figure S4.) demonstrated the presence of functional domains required for activity. In addition, gene expression data available through ORCAE showed that all identified *D. gallinae* miRNA components are universally expressed in adult male and female mites and all other life-stages.

### *Piwi* and *Argonaute-2* genes are absent in the *D. gallinae* genome

Searches were conducted to identify Argonaute and Piwi coding sequences in *D. gallinae* based on similarity to two Ago proteins (Ago1 and Ago2) and three Piwi proteins [aubergine (Aub), piwi, Ago3] from *T. urticae* and *Drosophila*. All *Ago* and *Piwi* sequence orthologues identified in *D. gallinae* met the criteria of reciprocal best-hit in *D. gallinae* and *T. urticae* genomes. Using this search methodology we identified 25 Ago orthologues in the *D. gallinae* genome, while no Piwi orthologues were identified (Table 1).

All functional Ago proteins contain two common structural features: a PAZ domain (responsible for small RNA binding) and a Piwi domain (responsible for catalytic activities) [35]. Therefore, in order to identify potential functional *D. gallinae* Ago proteins, all 25 *D. gallinae* Ago orthologues were analysed for domains using the Pfam database [27]. These searches identified 16 *D. gallinae* Ago orthologues that contained both the PAZ domain (Pfam: PF02170) and Piwi domain (Pfam: PF02171) (Additional file 6: Figure S4). In addition, all 16 *D. gallinae* Ago orthologues were found to have a DEDH catalytic slicer motif within each Piwi domain, indicating that these Ago orthologues likely retain slicer activity [36].

**Fig. 2:**
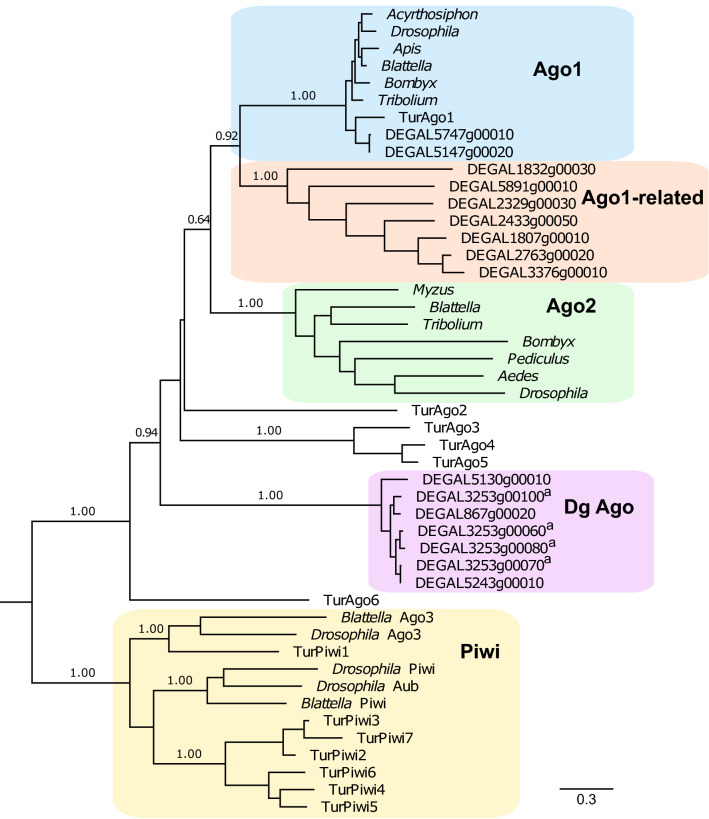
Phylogenetic analysis of Argonaute proteins (*Ago1*,* Ago2*) from *D. gallinae *and other arthropods. All *D. gallinae* sequences are available at ORCAE using the DEGAL accession numbers shown in the tree. The species and accession number of all other sequences are shown in Additional file 1: Table S1. All protein sequences were aligned using MUSCLE, and phylogenies were reconstructed using maximum-likelihood methods with a LG+G substitution model. Bootstrap support values > 0.6 from 1000 replicates are shown at each major node. Scale bar represents 0.3 substitutions per amino acid site. *D. gallinae *Argonaute genes that are located on the same genomic scaffold are indicated by a superscript letter

*Dermanyssus gallinae* Ago proteins with both PAZ and Piwi domains (a total of 16 *D. gallinae* Agos) were compared with orthologues from other arthropods, including the two-spotted spider mite, *T. urticae* (6 Ago orthologues and 7 Piwi orthologues) and insects (as detailed in Fig. 2). Our phylogenetic analysis identified two *D. gallinae* Ago1 orthologues (DEGAL5147g00020; DEGAL5747g00010) likely to be involved in the miRNA pathway. The remaining *D. gallinae* Ago orthologues belong to two major clades: the first containing seven members and closely related to Ago1 proteins (Fig. 2, Ago1-related clade); the second containing seven members and is unique to *D. gallinae* (Fig. 2; Dg Ago clade). Members of the Dg Ago clade show evidence of duplication, with four members (DEGAL3253g00060; DEGAL3253g00070; DEGAL3253g00080; DEGAL3253g00100) present as a tandem array on the same genomic scaffold. Strikingly, none of the identified *D. gallinae* Ago orthologues belong to either the Ago2 clade or Piwi clade (Fig. 2).

### RdRP is present in the *D. gallinae* genome

RNAi is a conserved gene silencing mechanism in eukaryotes. In non-animal eukaryotes RNAi-mediated gene silencing requires RdRP proteins [37]. However, among animals investigated to date, only nematodes require RdRP for RNAi-mediated gene silencing [37]. Using the *T. urticae* RdRP protein sequence as a query in the BlastP search against *D. gallinae* we identified nine RdRP orthologues in the *D. gallinae* genome that meet the criteria of reciprocal best hit in the *D. gallinae* and *T. urticae* genomes (Table 1). Based on gene expression data available through ORCAE, all nine *D. gallinae* RdRP orthologues are expressed and therefore may play a role in amplification and propagation of silencing signals (Table 1).

### Functional RNAi gene silencing in *D. gallinae*

**Fig. 3:**
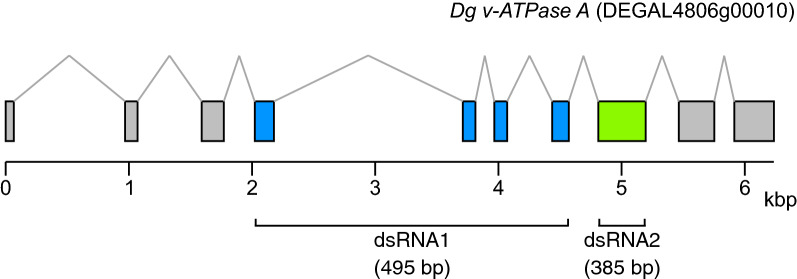
Regions of the *D. gallinae vATPase subunit A* (*Dg vATPase A*) gene used for dsRNA synthesis. Schematic representation of the *Dg vATPase A* gDNA locus in *D. gallinae* gDNA scaffold DEGAL4806g00010 (34.1 kbp) location of region 1 (R1) (exons 4, 5, 6 and 7) and region 2 (R2) (exon 8) used for dsRNA synthesis

**Fig. 4:**
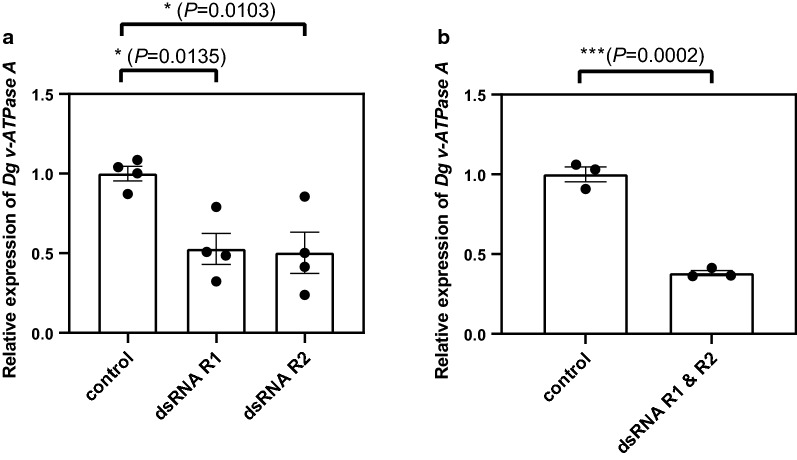
RNAi gene knockdown of *Dg vATPase A* in *D. gallinae*. Quantitative PCR gene expression analysis of *Dg vATPase A* expression in adult female *D. gallinae* mites fed on goose blood containing *lacZ* dsRNA (control) or *Dg vATPase A* dsRNA from R1 or R2. R1 and R2 *Dg vATPase A dsRNAs* were either added separately (**a**) or combined (**b**) in goose blood at a final concentration of 100 ng/μl, with the experiment shown in **b** containing equimolar amounts of R1 and R2 dsRNA. *Dg vATPase A* expression is shown at 96 h post-feed and is normalised to glyceraldehyde 3-phosphate dehydrogenase expression. Individual data points for biological replicates are shown with mean ± standard error of the mean (SEM) (*n* = 3–4). Asterisks represent significant difference at *P* < 0.05 between treatments determined by a one-way ANOVA with Dunnett’s multiple comparison test (**a**) or Student's* t*-test (**b**)

Two non-overlapping regions of the *Dg vATPase A* gene were selected for synthesis of dsRNA. Region 1 (R1: 495 bp, corresponding to exon 4–7) and region 2 (R2: 385 bp, corresponding to exon 8) were used for *in vitro* synthesis of dsRNA (Fig. 3). dsRNAs were incorporated into goose blood, which was subsequently provided to adult female *D. gallinae* mites using an *in vitro* feeding device. After feeding, engorged mites were selected for gene expression analysis based on a visible blood meal contained within the body, i.e. a bright red blood meal was clearly visible through the transparent cuticle in the engorged mites. Mites with no visible signs of feeding were discarded and not used for expression analyses. Feeding R1 and R2 dsRNA (both at 100 ng/μl) in separate feeding trials to adult female *D. gallinae* mites resulted in a significant 1.9-fold reduction (for both R1 and R2 feeding trials) in the expression of *Dg vATPase A* compared with control mites that fed on blood containing non-specific *lacZ* dsRNA (one-way ANOVA with Dunnett’s multiple comparison test: Control* vs* R1, *F*_(2, 9)_ = 8.303, *P* = 0.0135; Control* vs* R2,* F*_(2, 9)_ = 8.303, *P* = 0.0103) (Fig. 4a). Reproducibility of RNAi-mediated *Dg vATPase A* gene silencing, using two non-overlapping dsRNAs targeting the same gene, demonstrated specificity and therefore makes off-target effects unlikely. In addition, expression of the housekeeping gene *GAPDH* was comparable across treatment mites (*Dg ATPase A* dsRNA) and control mites (non-specific *lacZ* dsRNA), suggesting that there was no global (non-specific) change in transcription of *GAPDH* as a result of dsRNA delivery.

Under similar feeding methodology, combining R1 and R2 *Dg vATPase A* (100 ng/μl dsRNA, consisting of an equimolar mix of R1 and R2 dsRNA) dsRNAs, resulted in a significant 2.6-fold reduction in the expression of *Dg vATPase A* compared with control mites that fed from non-specific *lacZ* dsRNA (Student's* t*-test: *t*_(4)_ = 12.58, *P* = 0.0002) (Fig. 4b).

Due to the important cellular function of *Dg vATPase A* it might be expected that gene silencing would result in a physiological change in *D. gallinae*. We note that when a *vATPase A* ortholog is silenced in *T. urticae*, it results in a dark-body phenotype [23, 38], however a colour-change phenotype was not observed in *D. gallinae*.

### RNAi-mediated gene silencing in *D. gallinae* is initiated quickly and is long lasting

**Fig. 5:**
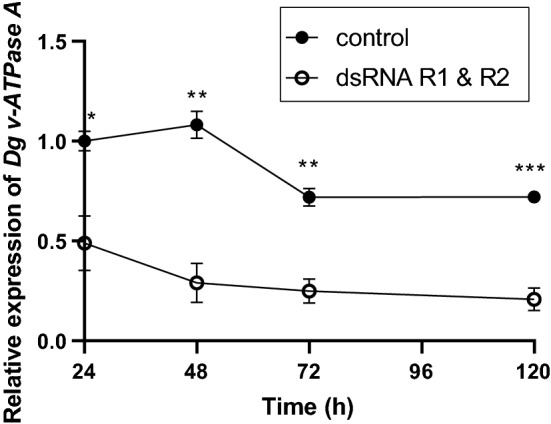
Persistence of RNAi gene knockdown of *Dg vATPase A* in *D. gallinae*. Quantitative PCR gene expression analysis of *Dg vATPase A* expression in adult female *D. gallinae* mites fed goose blood containing *lacZ* dsRNA (control) or combined *Dg vATPase A* dsRNA from R1 and R2. In both the control and *Dg vATPase A* dsRNA feeding experiments total dsRNA was delivered at 100 ng/μl (final concentration, containing equimolar amounts of R1 and R2 dsRNA), and *Dg vATPase A* expression levels were monitored over a 120-h post-feed time course. Individual data points for biological replicates are shown with mean ± SEM indicated (*n* = 3–4). Asterisks represent significant difference (*P* < 0.05) between treatments determined by Student's* t*-test

Feeding combined R1 and R2 *Dg vATPase A* dsRNA to adult female *D. gallinae* mites resulted in a rapid reduction of *Dg vATPase A* gene expression compared to that in control mites treated with the non-specific *lacZ* dsRNA. A significant reduction in *Dg vATPase A* expression was detectable by 24 h post dsRNA delivery, and the reduction was maintained for at least 120 h post dsRNA delivery (Fig. 5). At each time-point analysed, including 24, 48, 72 and 120 h, the expression of *Dg vATPase A* was significantly reduced by two-, 3.7-, 2.9- and 3.4-fold, respectively, relative to control (*lacZ*)-fed mites (Student's t-test: 24 h, *t*_(4)_ = 3.523, *P* = 0.0244; 48 h, *t*_(4)_ = 6.627, *P* = 0.0027; 72 h, *t*_(4)_ = 6.304, *P* = 0.0032; 120 h, *t*_(4)_ = 8.641, *P* = 0.0010) (Fig. 5).

### Oral delivery of siRNAs does not downregulate target gene expression

**Fig. 6:**
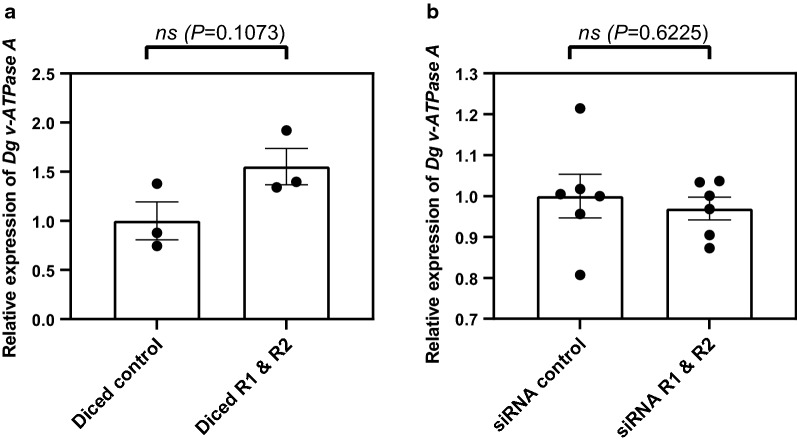
siRNAi gene knockdown of *Dg vATPase A* in *D. gallinae*. Quantitative PCR gene expression analysis of *Dg vATPase A* expression in adult female *D. gallinae* mites fed on *Dg vATPase A* siRNAs or *lacZ* siRNA (control).** a** Long R1 and R2 *Dg vATPase A* dsRNAs and long *lacZ* dsRNA were diced to produce short siRNAs and fed to adult female *D. gallinae* mites in goose blood (100 ng/μl).** b** siRNAs (27-mer) for *Dg vATPase A* and *LacZ* (control) were commercially synthesized and fed to adult female *D. gallinae* mites in goose blood (100 ng/μl). *Dg vATPase A* expression in both** a** and** b** is shown at 96 h post-feed and is normalised to *GAPDH* expression. Individual data points for biological replicates are shown with the mean ± SEM indicated (*n* = 3–6). No significant differences (*ns*) were detected between treatment groups using Student's* t*-test

Short dsRNAs were produced by either *in vitro* Dicer treatment of synthesized long R1 and R2 dsRNAs (method 1) or the commercial synthesis of two 27-bp dsRNAs corresponding to regions of the *Dg vATPase A* gene (method 2). The sequence of each synthesized siRNA is shown in Additional file 4: Figure S2. Feeding trials were used to deliver either diced-R1/R2 dsRNAs (Fig. 6a) or synthesized 27-bp dsRNAs (Fig. 6b), along with the appropriate control dsRNA to adult female *D. gallinae* mites. Neither diced-R1/R2 dsRNAs nor synthesized 27-bp dsRNAs resulted in downregulation of *Dg vATPase A* gene expression, and treated mites had comparable expression levels with control fed mites (Student's *t*-test: diced-R1/R2 dsRNAs, *t*_(4)_ = 2.069, *p* = 0.1073; synthesized 27-bp dsRNAs, *t*_(10)_ = 0.5080, *P* = 0.6225] (Fig. 6a, b).

## Discussion

The results of this study demonstrate the presence of genes encoding components of two of the main RNAi pathways in the genome of *D. gallinae*, namely the siRNA and miRNA pathways. Both exogenous and endogenous siRNA pathway components were identified in the *D. gallinae* genome, but the major piRNA components, the *Piwi*, *Aub* and *Ago3* genes, were lacking, indicating either very dissimilar sequences of these genes or, more probable, the absence of this pathway in *D. gallinae*. We also demonstrate the utility of an *in vitro* feeding methodology [33] for the successful delivery of dsRNA to initiate prolonged, gene-specific RNAi in *D. gallinae* but show that orally delivered short siRNA failed to initiate RNAi for the gene investigated herein.

In arthropods, three main RNAi pathways co-exist, namely the siRNA pathway, the miRNA pathway and the piRNA pathway [17]. To date, in the few mite species that have been studied, only *T. urticae* has been found to retain all three pathways [25], while in other species of mite the piRNA pathway is notably absent. The piRNA pathway appears to be absent in the sheep scab mite; *Psoroptes ovis* [20]; the human itch mite, *Sarcoptes scabiei* [19]; the house dust mites, *Dermatophagoides farinae* [39], *D. pteronyssinus* [19] and *Euroglyphus maynei *[19]. In *Drosophila*, where the piRNAi biology is best characterised, the piRNA pathway silences transposons during germline development, thereby protecting the inherited genome from mutation [40]. The apparent lack of the piRNA pathway in *D. gallinae* and some other members of the Acari, compared with the retention of this pathway in *T. urticae*, reflects the dynamic nature of RNAi pathways in mites and may indicate species-specific biology. As more mite genomes are sequenced, comparative approaches comparing transposon load in mites with and without functional piRNA pathways will likely yield important insights into mite control of transposable elements. Currently, the molecular mechanism(s) which protect *D. gallinae* and other Acari lacking the piRNA pathway against the deleterious effects of transposon activity in the germline await investigation.

This study demonstrates that components of the siRNA pathway are present in the *D. gallinae* genome. One notable absence was the lack of a *D. gallinae* Ago2 orthologue, onto which the mature siRNA, required for gene silencing, is loaded (Fig. 1). Although a definitive orthologue of Ago2 was not discovered in *D. gallinae*, the argonaute family was expanded with 25 family members, some of which are unique to *D. gallinae*, raising the possibility that, in *D. gallinae, Ago2* is replaced by another yet uncharacterised argonaute. Our experimental work presented here confirms that although an Ago2 orthologue is missing in *D. gallinae*, the siRNA pathway is functional and can be exploited by feeding exogenous dsRNA, resulting in specific knockdown of the targeted *Dg vATPase A* gene. In our RNAi feeding experiments, *Dg vATPase A* was chosen as a target, as it has been previously targeted in *T. urticae*, where downregulation resulted in a dark-body phenotype in *vATPaseA* gene-silenced *T. urticae* mites [23, 38]. Using a similar dsRNA feeding methodology, we achieved gene silencing of *Dg vATPase A*, but this was not associated with a notable colour change phenotype. However, for haematophagous mites, such as *D. gallinae*, colour change phenotypes are particularly difficult to determine due to the intense red colour of the mites after blood-feeding.

In our experiments investigating dsRNA length, we were able to achieve robust and reproducible *Dg vATPase A* knockdown using dsRNAs of 385 and 495 bp. However, when we targeted the same gene with short siRNAs, produced from either dicing the long dsRNAs or commercially synthesizing a short 27-bp siRNA, the gene knockdown effect was lost. Recent dsRNA feeding experiments in *T. urticae* demonstrate that there is a size threshold for effective gene silencing, with long dsRNAs resulting in robust gene silencing and shorter dsRNAs (100–200 bp) being ineffective [38]. In *D. gallinae*, the lower limit of dsRNA size for efficient gene silencing is currently unknown and awaits further investigation. The lack of gene silencing by short siRNAs in *D. gallinae* may result from three possibilities: (i) uptake of short dsRNA from food is limited and therefore does not induce RNAi pathway; (ii) the abundance of siRNAs saturates the intracellular RNA uptake system; or (iii) short dsRNAs are transported but not recognized by the cellular RNAi processing machinery. To investigate these possibilities, we are planning to study the delivery of siRNA by microinjection in *D. gallinae*. Importantly, short 21-bp siRNAs efficiently silence *Distal-less* (*Dll*) in *T. urticae* when microinjected into females, resulting in a transfer of siRNA to oviposited eggs and loss-of-function phenotype of the target gene [18].

Functional RNAi is an important tool in both model and non-model organisms to investigate genes of unknown function. For example, RNAi has been exploited in *Drosophila* to investigate gene function in cultured cells using high-throughput screening methods [41]. Functional RNAi in *D. gallinae*, using the methodologies described here, is particularly timely for investigating genes of unknown function in *D. gallinae*. Recent completion of the draft *D. gallinae* genome sequence (959 Mbp assembly) identified 14,608 protein coding genes, of which 768 appear to be unique to *D. gallinae*, without similarity to proteins in the NCBI nucleotide protein database [13]. Therefore, the development of a robust and reproducible RNAi methodology, coupled with -omic technologies, offers a powerful approach to begin investigating gene function and biology that is unique to *D. gallinae*. In addition, RNAi methodologies for *D. gallinae* are likely to be a useful tool in the development of novel control methods, including vaccine development, as such novel methods will support the research community with interests in *D. gallinae* control [42–44]. RNAi-mediated gene silencing in *D. gallinae*, coupled with either *in vitro* bioassays [33] or on-bird feeding assays [32], will allow rapid screening of potential *D. gallinae* vaccine candidates and druggable targets. Significantly, utilising RNAi to screen for vaccine candidates in *D. gallinae* will likely speed up the process of antigen discovery and in turn conform to the 3R principles (replacement, reduction and refinement) of using fewer animals for vaccine trials.

## Conclusions

We found evidence for the presence of two RNAi pathways in *D. gallinae* and also successfully demonstrated that our functional gene knockdown protocol can be initiated by feeding gene-specific dsRNA in an improved *in vitro* feeding device. This opens the door for larger scale, genome-wide screening for novel *D. gallinae* control targets and also provides the opportunity to ascribe functions, using phenotypic assays, to the multiple genes of unknown function identified within the *D. gallinae* genome, for which no homologues exist in other species.

## Supplementary Information

**Additional file 1: Table S1.** Ago sequence accession numbers used for phylogenetic reconstruction.

**Additional file 2: Figure S1.** Alignment of mite vATPase A proteins.

**Additional file 3: Table S2.** qPCR and RNAi construct primer sequences.

**Additional file 4: Figure S2.** Regions used for synthetic siRNA synthesis.

**Additional file 5: Figure S3.** Domain architecture of *D. gallinae* Dicer proteins.

**Additional file 6: Figure S4.** Domain architecture of *D. gallinae* argonaute proteins.

## Data Availability

The *Dg vATPase A* nucleotide coding sequence is available in the NCBI database using the following accession number: MW032475.

## Abbreviations

Ago: Argonaute
Aub: Aubergine
BLAST: Basic local alignment search tool
cDNA: Complementary DNA
Dcr-1: Dicer-1
Dcr-2: Dicer-2
*Dg vATPase A*: *D. gallinae* vacuolar ATPase subunit A
dsRNA: Double-stranded RNA
EDTA: Ethylenediaminetetraacetic acid
EU: European Union
GAPDH: Glyceraldehyde 3-phosphate dehydrogenase
gDNA: Genomic DNA
Loqs: Loquacious
miRNA: MicroRNA
ML: Maximum likelihood
PASHA: Partner of Drosha
piRNA: Piwi-interacting RNA
PIWI: P-element-induced wimpy testis
R1: Region 1
R2: Region 2
RdRP: RNA-dependent RNA polymerase
RISC: RNA-induced silencing complex
RNAi: RNA interference
siRNA: Small interfering RNA
TAE: Tris-acetate-EDTA
XPO5: Exportin-5
Zuc: Zucchini
