# Supplementary material for: RNAi gene knockdown in the poultry red mite, Dermanyssus gallinae (De Geer 1778), a tool for functional genomics
Source: Parasit Vectors. 2021 Jan 18;14:57. doi: 10.1186/s13071-020-04562-9 (PMC7813172; doi:10.1186/s13071-020-04562-9)
Supplement: Supplementary file 2 — Additional file 2: Figure S1. Alignment of mite vATPase A proteins. [file 13071_2020_4562_MOESM2_ESM.docx]

**Additional file 2: Figure S1. Alignment of mite vATPase A proteins.** Dg vATPase A and closely related vATPase A from mites were aligned using MUSCLE. Conserved regions in all sequences are highlighted (*). Sequences included in the alignment are: Dg vATPase A (DEGAL4806g00010); and vATPase A from the following mites: *Varroa destructor* (XP_022670784); *Tropilaelaps mercedesae* (OQR76956) and *Galendromus occidentalis* (XP_003741079). Conserved regions, against which coding sequence amplification primers were designed are highlighted in red.
