# Supplementary material for: RNAi gene knockdown in the poultry red mite, Dermanyssus gallinae (De Geer 1778), a tool for functional genomics
Source: Parasit Vectors. 2021 Jan 18;14:57. doi: 10.1186/s13071-020-04562-9 (PMC7813172; doi:10.1186/s13071-020-04562-9)
Supplement: Supplementary file 4 — Additional file 4: Figure S2. Regions used for synthetic siRNA synthesis. [file 13071_2020_4562_MOESM4_ESM.docx]

**Additional file 4: Figure S2. Regions used for synthetic siRNA synthesis.**
