# Supplementary material for: RNAi gene knockdown in the poultry red mite, Dermanyssus gallinae (De Geer 1778), a tool for functional genomics
Source: Parasit Vectors. 2021 Jan 18;14:57. doi: 10.1186/s13071-020-04562-9 (PMC7813172; doi:10.1186/s13071-020-04562-9)
Supplement: Supplementary file 5 — Additional file 5: Figure S3. Domain architecture of D. gallinae Dicer proteins. [file 13071_2020_4562_MOESM5_ESM.docx]

**Additional file 5: Figure S3. Domain architecture of *D. gallinae* Dicer proteins.** For comparison the domain architecture of *D. melanogaster* dicer-1 (Dcr-1) and dicer-2 (Dcr-2) shown. *D. gallinae* Dcr-1 and Dcr-2 were identified as orthologues of Drome Dcr-1 and Dcr-2, respectively. Pfam (https://pfam.xfam.org) functional domains include: Helicase_C (Helicase conserved C-terminal domain, PF00271); Dicer_dimer (Dicer dimerisation domain, PF03368); PAZ (PAZ domain, PF02170); Ribonuclease_3 (Ribonuclease III domain, PF00636); DEAD (DEAD/DEAH box helicase, PF00270). The length of each protein is shown as number of amino acids.
