# Supplementary material for: RNAi gene knockdown in the poultry red mite, Dermanyssus gallinae (De Geer 1778), a tool for functional genomics
Source: Parasit Vectors. 2021 Jan 18;14:57. doi: 10.1186/s13071-020-04562-9 (PMC7813172; doi:10.1186/s13071-020-04562-9)
Supplement: Supplementary file 6 — Additional file 6: Figure S4. Domain architecture of D. gallinae argonaute proteins. [file 13071_2020_4562_MOESM6_ESM.docx]

**Additional file 6: Figure S4. Domain architecture of *D. gallinae* argonaute proteins.** For comparison the domain architecture of *D. melanogaster* argonaute-1 (Drome Ago1) is shown. *D. gallinae* argonaute proteins were identified as orthologues of Drome Ago1 and are ranked in order of best blast hit to Drome AGO1. Pfam (https://pfam.xfam.org) functional domains include: ArgoN (N-terminal domain of argonaute, PF16486); ArgoL1 (argonaute linker 1 domain, PF08699); PAZ (PAZ domain, PF02170); ArgoL2 (argonaute linker 2 domain, PF16488); ArgoMid (mid domain of argonaute, PF16487); Piwi (Piwi domain, PF02171). Genes located on the same *D. gallinae* scaffold are highlighted using the superscript letters a-e. The length of each protein is shown as number of amino acids. No Pfam domains were detected (nd, none detected) for DEGAL5147g00040.
